# Supplementary material for: An image quality assessment index based on image features and keypoints for X-ray CT images
Source: PLoS One. 2024 Jul 11;19(7):e0304860. doi: 10.1371/journal.pone.0304860 (PMC11238976; doi:10.1371/journal.pone.0304860)
Supplement: Attachment — Submitted filename: Response letter_PONE-D-23-42003_R2.docx [file pone.0304860.s001.docx]

Dr. Yan Chai Hum

Academic Editor

PLOS ONE

Manuscript Number: PONE-D-23-42003R1

Dear Dr. Yan Chai Hum,

Thank you so much for your e-mail and review of the manuscript (PONE-D-23-42003R1) that we sent on 5 March 2024. We thank reviewers for providing constructive and helpful comments regarding the improvement of the original manuscript, “Image quality assessment index based on image features and keypoints for X-ray CT images”.

Here, we are sending files of our revised manuscript. All changes have been made in response to the reviewers’ suggestion, and itemized response to the individual reviewer’s comments are also attached as below.

**Reviewer #2:**

**- In the introduction section, include some other research applications on medical imaging as literature. Some suggestions are: Multi-modal medical image fusion framework using co-occurrence filter and local extrema in NSST domain; An end-to-end content-aware generative adversarial network based method for multimodal medical image fusion; TSJNet: A Multi-modality Target and Semantic Awareness Joint-driven Image Fusion Network; Clustering based Multi-modality Medical Image Fusion; Directive clustering contrast-based multi-modality medical image fusion for smart healthcare system.**

Thank you for your suggestions. I deeply appreciate you providing various previous research on image fusion.

While these seem to be significant papers for applications on medical imaging, there appeared to be a slight mismatch with our research theme focusing on image quality evaluation. Therefore, following your suggestion, we surveyed recent research in the field of medical imaging once again, particularly in image quality assessment and analysis, and substantially improved the explanation in the Introduction section by including literature to emphasize the significance of our study.

We have reflected this comment by lines 28–40.

**- The authors should test their model with their dataset (not only from the public dataset). The complexity comparison should be made. Cross-Validation is lacking in this paper.**

Thank you for your valuable feedback.

We recognize that since our proposed method is not based on such as a deep-learning model, general cross-validation methods cannot be directly applied. Also, analysis data used in this study were acquired by us (the dataset has been made publicly available as follow the request of PlosOne).

However, to assess the reliability and utility of our proposed method, we have conducted comparisons across various aspects as much as possible. While it may be necessary to validate the results across different modalities and tasks to generalize them further, these ideas have been detailed as limitations ( lines 334–346).

Regarding the assessment of complexity, we have added the measurement results to Table 3 (lines 233–245).

**- For the validation and best performance of result, use graphical analysis like histogram or intensity profile analysis.**

We appreciate the interesting suggestion.

Our proposed method does not output generated images, but rather obtains quantitative values. Visualization of feature points also involves simply drawing points and lines on the original image, so the image itself is not transformed in any way. Therefore, please understand that graphical analysis such as histograms or intensity profile analysis cannot be directly applied to verify the results.

However, we acknowledge the importance of using appropriate visual techniques to understand and interpret the results effectively.

Therefore, we employ several graphs and tables to express the results more clearly. These figures are intended to complement the quantitative results and make it easier to visually understand the performance and effectiveness of the proposed method. Therefore, in view of the fact that the proposed method obtains quantitative values, we conduct appropriate graphical analysis in the validation of the results and utilize them to explain the effectiveness and performance of the proposed method in more detail.

**- Rewrite conclusion. Add concluding, contribution points in it briefly. Abstract needs to rewritten with better summary of paper.**

Thank you for your advice.

I have rewritten the conclusion section to make the contributions of this study clearer. Additionally, I have revised the entire abstract to ensure a more appropriate summary of the paper.

**Reviewer #3:**

**- The introduction does not make clear what the application context requires of the method. The reason why evaluation indicators such as SSIM cannot be used and new indicators need to be proposed is not well reflected in the introduction.**

Thank you for your important suggestions.

To clarify what the proposed method requires in the context of the application, we have improved the entire introduction and revised it to provide a logical progression. In particular, we have tried to reflect more clearly the situations in which existing evaluation metrics are not applicable and why it is necessary to propose new metrics. Furthermore, I have pointed out the situations where existing evaluation indicators do not function properly in the context of specific applications and clearly reflected why the proposal of new indicators is necessary.

We have reflected this comment by lines 28–59.

**- The design of the experimental section is problematic. The comparison of algorithms in the experimental section does not reflect well the superiority of the proposed quality evaluation method. At the same time, the algorithm lacks data on time complexity.**

Thank you for your feedback.

Acknowledging the objective fact that the results of SSIM are widely accepted as an image quality evaluation index, this verification assumes that the SSIM is evaluated as an external standard.

The ability of the proposed metric to capture image quality changes similar to the SSIM can be demonstrated through its correlation with the SSIM. By clarifying it, we can demonstrate the validity and reliability of the proposed index. On the other hand, by demonstrating the robustness of the proposed index against transformations such as translation, rotation, and scaling, we assert its superiority and usefulness over the SSIM. This can be argued by showing that the SSIM changes in value for these transformations, but the proposed index does not change (i.e., is not correlated with SSIM). Therefore, although we cannot agree the reviewer's suggestion that there is a problem with the design of this validation, we have confirmed the description in the Methods section to make our arguments clearer.

Additionally, we have included data on the time complexity of the proposed method in Table 2. This makes the efficiency and practicality of the proposed method clear (lines 233-245).

**- Insufficient arguments for the conclusions drawn from the experimental results. The conclusion in line 254 that the scores obtained by the algorithmic metrics proposed after the images have undergone affine transformations are not sufficiently correlated with SSIM to indicate that the algorithms are robust is not very convincing. Consideration could be given to validating this point based on the correlation between the results of the algorithm after the affine transformation and the results of algorithms such as CW-SSIM and the like (correlation algorithms that can still efficiently detect unstructured distortions).**

Thank you for your feedback from the insightful perspective.

Considering that the CW-SSIM is limited in its ability to function only under certain conditions, such as when the amount of transformation, scaling, and rotation is small compared to the size of the wavelet filter, under the conditions verified in this study, it is speculated that it do not exhibit sufficient robustness compared to the proposed index. However, this idea seems interesting and important, and we plan to validate it by comparing our method with the CW-SSIM based on the findings of this study. Additionally, we have added information about the CW-SSIM in the discussion section and included sentences to further clarify the significance of this research.

We have reflected this comment by lines 350–353.

**- There is a lack of explanation about the reason for choosing the feature extraction algorithm. Whether the specific characteristics of CT images were taken into account or whether there were other considerations.**

Thank you for your feedback.

To enhance the versatility of the proposed metrics, we considered it appropriate to select more general algorithm rather than methodologies tailored to specific imaging tasks (such as considering specific characteristics of CT images) to capture a wide range of features. Therefore, we have added details on the rationale for selecting the feature extraction algorithm in lines 108–118.

I hope this adequately addresses the reviewer's requirements.

Again, thank you for giving us the opportunity to strengthen our manuscript with your valuable comments and queries. We believe that we have properly addressed reviewers’ comments and hope that the revised manuscript is now acceptable publication in PLOS ONE. However, we would be glad to respond to any further questions and comments that you may have.

Thank you for your generous consideration.

Sincerely yours,

Sho Maruyama

Department of Radiological Technology,

Gunma Prefectural College of Health Science

E-mail. maruyama@gchs.ac.jp
